# Strategies for reducing speckle noise in digital holography

**DOI:** 10.1038/s41377-018-0050-9

**Published:** 2018-08-01

**Authors:** Vittorio Bianco, Pasquale Memmolo, Marco Leo, Silvio Montresor, Cosimo Distante, Melania Paturzo, Pascal Picart, Bahram Javidi, Pietro Ferraro

**Affiliations:** 1 CNR-ISASI Istituto di Scienze Applicate e Sistemi Intelligenti “E. Caianiello”, via Campi Flegrei 34, 80078 Pozzuoli (NA), Italy; 2 Université du Maine, CNRS UMR 6613, LAUM, Avenue Olivier Messiaen, 72085 Le Mans Cedex 9, France; 3 Electrical and Computer Engineering Department, University of Connecticut, U-4157, Storrs, CT 06269 USA

## Abstract

Digital holography (DH) has emerged as one of the most effective coherent imaging technologies. The technological developments of digital sensors and optical elements have made DH the primary approach in several research fields, from quantitative phase imaging to optical metrology and 3D display technologies, to name a few. Like many other digital imaging techniques, DH must cope with the issue of speckle artifacts, due to the coherent nature of the required light sources. Despite the complexity of the recently proposed de-speckling methods, many have not yet attained the required level of effectiveness. That is, a universal denoising strategy for completely suppressing holographic noise has not yet been established. Thus the removal of speckle noise from holographic images represents a bottleneck for the entire optics and photonics scientific community. This review article provides a broad discussion about the noise issue in DH, with the aim of covering the best-performing noise reduction approaches that have been proposed so far. Quantitative comparisons among these approaches will be presented.

## Digital holography: Tackling speckle

Digital holography has become an important and popular imaging technology with applications in biomedicine, metrology and 3D displays. However, its coherent nature means that it suffers from speckle, an undesirable source of noise that degrades the quality of holographic images. In a Review of the topic, Vittorio Bianco and coworkers now discuss the details of the problem and the various optical and numerical solutions that have been developed to combat the issue. Optical approaches discussed include the use of optical sources with either reduced or no coherence such as white light and random lasers or alternatively employing rotating or moving diffusers. In addition, a wide variety of denoising algorithms and digital spatial filtering methods have also been successfully developed to reduce the appearance of speckle.

## Introduction

Image denoising is a highly investigated research field in digital coherent imaging^1,2^. Improving image quality is important for all digital systems that provide images that have been degraded by several types of artifacts. Depending on light sources, sensor resolution or imaged scene sharpness, obtaining high-quality images can be very challenging. One of the most fascinating technologies for three-dimensional (3D) coherent imaging to emerge in the past two decades is digital holography (DH)^3–11^. DH has the unique property of retrieving the entire complex wavefront of a recorded object, thereby allowing the measurement and manipulation of both amplitude and phase information^11^. The incredible technological development of digital sensors and optical elements has made DH the primary approach in several research fields, such as quantitative phase 3D imaging of biological samples^12–17^, microfluidics^18,19^, optofluidics^20,21^, 3D particle tracking^22,23^, 3D optical display^24–26^, homeland security^27^, optical security and encryption^28^, cultural heritage^29^, and compressive holographic imaging^30,31^.

However, holographic image quality is severely degraded by undesired artifacts due to the coherent nature of the light sources, thereby resulting in images that are corrupted by a mixture of additive uncorrelated noise and the so-called speckle^2^. Therefore, despite the benefits and opportunities that DH imaging offers, the noisy nature of holographic images limits the development of all aforementioned applications. This has encouraged many research groups to work on noise reduction in DH, and many solutions, both numerical and optical, have been proposed for overcoming this problem^32–85^.

Considering the large variety of methods that have been introduced so far for tackling the problem of denoising in coherent imaging systems, such solutions need to be classified according to the attributes that they share. In this sense, we divide the methods into two main classes: (i) methods that rely on engineering the laser source and (ii) techniques that, by using a conventional high-coherence source, aim at optimizing either data recording or numerical reconstruction processes. In the second category, we span both methods that adopt an optimized acquisition scheme (e.g., set-ups that introduce noise diversity among multiple recordings) and methods that transfer complexity from the recording system to the numerical reconstruction algorithms. In the second framework, we can distinguish between Bayesian approaches, which rely on prior information on the noise statistics, and non-Bayesian techniques. For all the above-mentioned categories, a wider classification can be performed among methods that are specifically suited for denoising the amplitude reconstruction, the phase-contrast map, or both by operating on the numerical complex wavefront. Different from the general strategies of image processing, the methods that we will overview must satisfy one main constraint: they must operate while preserving the coherence between amplitude and phase, thereby preserving all the features of a digital hologram. This requirement significantly differentiates the problem of denoising coherent datasets from the issue of denoising the sole amplitude image and makes it more complex to handle. This also justifies the large number of efforts in this field.

This review is divided into the following sections. First, we will describe the noise formation process in DH and consider causes, limitations, and possible solutions. In the third section, we will introduce the denoising process by considering various strategies. In particular, with the aim of providing readers with a detailed and clear overview of all the approaches that are followed by research groups worldwide, the described methods will be classified according to their main adopted strategies: optical or numerical. Among the latter, Bayesian or non-Bayesian approaches will be identified, focusing on non-Bayesian spatial filtering. Finally, we will divide such methods between those that are applied for amplitude or phase enhancement. In some cases, the strategy includes one or more of these attributes. Through this taxonomy, almost all the reviewed techniques may be identified. It will be shown how advantages and drawbacks of each technique strictly depend on the classes to which it belongs and how the hybrid approaches are emerging as the most effective in tackling the denoising problem.

## Noise process in DH

Noise in digital holograms has several origins that are related to the so-called “technical” noise and the coherent noise. The technical noise is the standard noise that is encountered in all photonic systems that detect light and convert it into digital data. As a general rule, the noise sources that contribute to the technical noise are the photon noise, the electronic noise from the recording sensor, and the quantization noise due to the analog-to-digital conversion. Several authors have discussed these noise sources in digital holographic images. In 2005, Mills et al. evaluated the quality of reconstructed images for various quantization levels^77^. They concluded that the object phase distribution does not substantially influence image appearance above the threshold of 4-bit quantization and that at bit depths <4 bits, random phase modulation introduces a speckle noise effect. More recently, in 2011, Pandley and Hennelly studied the influence of quantization error in recorded holograms on the fidelity of both the intensity and phase of the reconstructed image^78^. They showed that quantization error is introduced as uniformly distributed additive noise into the recording plane and this manifests itself as a complex noise in the reconstruction plane with Gaussian-distributed real and imaginary parts, Rayleigh-distributed amplitude and uniformly distributed phase. In 2011, Picart et al. described, from both theoretical and experimental points of view, the noise that appears in holographic images when holograms include saturation, up to 90%, due to the finite number of bits of the sensor^46^. These previous works focused mainly on the quality of the image amplitude that was reconstructed from the digitally recorded hologram. However, the quantum nature of light interferes with the photon noise. Gross’s group demonstrated that the ultimate noise in amplitude reconstruction is dominated by the photon noise^86–89^. Particularly, they studied the influence of the noise sources in heterodyne holography. To reach the shot-noise ultimate limit, they proposed a hybrid digital processing approach that combines the phase shift method and the spatial Fourier transform to reduce artifacts that are due to parasitic orders and phase shift nonlinearity.

Coherent noise is a very particular noise that is only encountered in coherent imaging when imaging with light sources that have a large coherence length, such as single-longitudinal-mode lasers. Such lasers may have coherence lengths of several tens of meters. It follows that the nature of the surface (or thickness) of the sample may generate a speckle pattern when recording digital holograms. This effect is easy to understand: each “point” at the surface of the object emits a wavelet onto the sensor and this wavelet has a random phase because of the surface roughness. At the detector plane, all emitted wavelets are coherently mixed and produce a phenomenon of multiple random beam interferences. Thus the object wave at the detector plane is a speckle wave. Moreover, the special character of coherent noise, as partly signal-dependent noise, requires different approaches for filtering, as in case of simple additive noise^90^. The statistics of such speckle pattern were described in the past and can be found in several books^2,8,89–93^. They include first-order statistics such as the probability density functions of the amplitude, intensity, and phase and the average value and standard deviation of the intensity. The second-order statistics are useful for describing the structure of the speckle field, especially the correlation length in the three directions of a set of reference coordinates that are attached to the observation plane.

Basically, a digital hologram is obtained by the coherent mixing of a so-called object wave (*O*), diffracted in the recording plane by the object, which is located at distance *d*_0_ from this plane, and a reference wave (*R*). This interference pattern is recorded with a pixel matrix sensor and can be mathematically expressed as the following Eq. (1)^7–9,^^94–96^:

1 $$H = \left| R \right|^2 + \left| O \right|^2 +\, R^ \ast O + RO^ \ast$$

As a general rule, the reference wave is smooth and has a tilted plane wavefront (“off-axis” holography). If the reference wavefront is not tilted, the configuration is said to be “in-line”, and phase shifting techniques^7^ are required to extract the so-called +1 order (term R*O in Eq. (1)) from the hologram. It follows that digital holograms of naturally rough objects are constituted by a speckle pattern that is modulated by micro-fringes, which originate from the coherent interference between the speckle field and the reference wave (which should be unspeckled). The main drawback is that the speckle, which is very useful for encoding the complex object field, becomes a strong enemy in the reconstructed image for both amplitude and phase recovery because it affects the quality of the reconstructed field and appears as parasitic noise. Practically, the reconstructed amplitude image from objects that have a rough surface is not uniform and exhibits dark and bright grains due to the speckle phenomenon (even if the initial object surface exhibits uniform brightness). The most important feature in the reconstructed image plane is the average size of the speckle grains, which is related to the transverse correlation length of the speckle field. As a general rule, the size of the speckle grain is linked to the physical parameters of the optical set-up^7–10,26^ according to Eq. (2):

2 $$\Delta x = \frac{{\lambda d_0}}{{Np_x}}$$

in which *λ* is the wavelength of light, *d*_0_ is the physical distance between the object and the sensor, *p*_*x*_ is the pixel pitch of the sensor, and *N* is the number of pixels along the *x*-direction. A similar relation holds for the *y*-direction.

Statistics of the speckle in the hologram plane can be determined by considering that the speckle field that is generated by the object surface is a random Gaussian process with zero mean. In the off-axis configuration, the speckle grain covers several pixels in the recording plane. Typically, 1 grain covers 3–4 pixels (or maybe more), so that the speckles can be considered “resolved speckles”. In the on-axis configuration, 1 grain is approximately 1 pixel; therefore, one deals with “unresolved speckles”. In the case of a resolved speckle, the statistics of the +1 order in the hologram plane follow a negative exponential for the intensity (*I* = |R*O|^2^) and the Rayleigh law for the amplitude (*a*_0_ = |R*O|). The digitally reconstructed image follows the same statistics of the speckle pattern in the hologram plane.

**Fig. 1:**
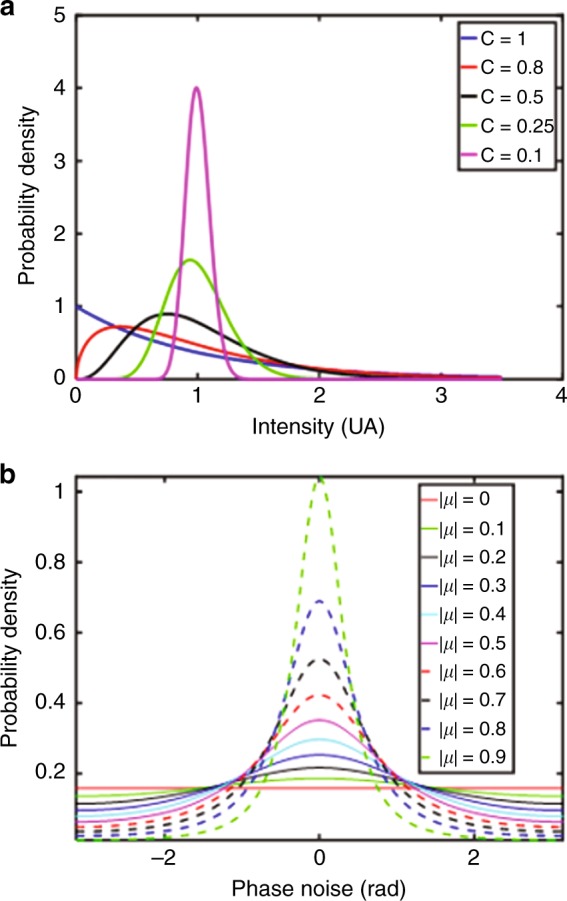
Statistical analysis of noise in DH. **a** Probability density of I versus values of the speckle contrast. **b** Curves of Eq. (3) for various values of the coherence factor. Adapted with permission from ref.^47^ [OSA The Optical Society]

Figure 1a shows the probability density of the intensity versus values of the speckle contrast, which is expressed as *C* = *σ* / 〈 *I* 〉, where *σ* is the standard deviation and 〈⋯〉 is the average operator. When *C* = 1, the probability density of intensity follows the negative exponential. When *C* decreases, which means that the speckles are averaged, then the probability density moves progressively from the negative exponential to a Gaussian-type curve (case *C* = 0.1). This means that when averaging the speckles in an image, the residual noise tends to a Gaussian noise, whereas this is not the case for the raw speckles.

When using DH for quantitative measurements, a “reference” phase is subtracted from the object phase. Since the phase difference is estimated modulo 2π, phase unwrapping is often required^97,98^. It follows that a speckle phase decorrelation occurs between the two states of the object and induces a noise in the phase variation between them, which is referred to as speckle phase decorrelation noise and denoted as *ε*^2,91,92^. Its probability density function is related to the modulus of the complex coherence factor, which is denoted as *μ*, between the two speckle fields and represents their correlation extent. With $\beta = \left| \mu \right|{\mathrm{cos}}\left( \varepsilon \right)$, the second-order probability density of the phase noise *ε* can be expressed as follows^2,91,92^:

3 $$p\left( \varepsilon \right) =	 \frac{{1 - \left| \mu \right|^2}}{{2\pi }}\left( {1 - \beta ^2} \right)^{ - 3/2} \\ 	\left( \!{\beta \sin ^{ - 1}\beta + \frac{{\pi \beta }}{2} + \sqrt {1 - \beta ^2} } \right)$$

Figure 1b shows curves of Eq. (3) that are obtained using various values of *μ*. If $\left| \mu \right| = 0$, the two fields are not correlated, and their phase difference is uniformly distributed over the interval [−*π*,*π*]. This is the worst-case condition for the phase noise. In contrast, if $\left| \mu \right| = 1$, the fields are fully correlated and the phase difference is close to 0. Noise is weak, and the probability density is narrower, although not completely Gaussian^47,48,99,100^.

This decorrelation noise may have several origins^7,9,10,46,48,94–97,99–101^. These are briefly reviewed in ref.^47^. Several authors proposed a large variety of techniques for reducing speckle noise, which can be classified into two main categories: optical methods^32,33,35,49–58^ and image processing methods^31,36,39,41,59–63^. Usually, to preserve the 2π phase jump in the wrapped phase map, reduction of decorrelation noise is processed on the sine and cosine images that are calculated from the raw phase^102,103^.

## Optical solutions for noise reduction

Noise reduction in DH has stimulated the work of several research groups worldwide. Among the huge variety of proposed strategies, one can identify two main categories of denoising approaches: optical and numerical methods. Optical techniques for noise reduction in DH mainly involve the reduction of the light coherence, engineering the light sources in the recording stage, or recording multiple holograms of the same object (i.e., the *looks*), which are captured under various realizations of the noise process. One can refer to the first case as *partially coherent illumination* (PCI)^104–108^. Moreover, synthetic aperture imaging can be viewed as an alternative way to mitigate the noise effect by modulating speckles from intervening optical elements or scattering layers^109,110^.

**Fig. 2:**
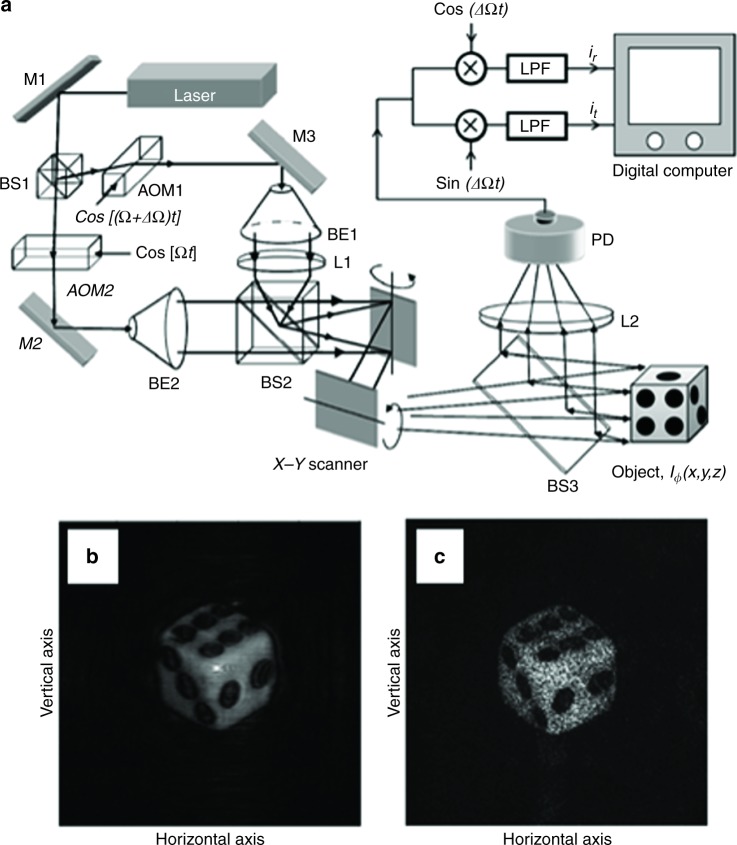
Optical Scanning Holography. **a** OSH set-up (M mirror AOM1, 2 acousto-optic modulators, BS1, 2, 3 beam splitters, BE1, 2 beam expanders, L focusing lens, x electronic multiplexer, LPF low-pass filter). **b** Numerical reconstruction of the complex hologram (free of speckle noise). **c** CCD imaging of a coherently illuminated dice. Reprinted with permission from ref.^105^ [OSA The Optical Society]

The feasibility of DH for PCI was first demonstrated by Poon in ref.^104^, where optical scanning holography (OSH) is proposed. The incoherent mode of OSH makes it possible to record a complex hologram without speckle noise^105^. Figure 2a reports the OSH set-up that is used to record a complex hologram of a diffusely reflecting object (in ref.^104^, the authors used a dice), while Fig. 2b, c show the numerical reconstructions of the recorded complex hologram (free of speckle noise) and the charge-coupled device imaging of a coherently illuminated dice, respectively.

More recently, Kim proposed a full-color holographic recording of outdoor scenes under natural daylight illumination, which was dubbed full-color self-interference incoherent DH^106^, i.e., neither lasers nor other special illuminations are used for recording or reconstruction. Using a simple optical set-up with a color camera and straightforward algorithms, holographic images are recorded and reconstructed under natural-light illumination and with full-color rendering, thereby removing the speckles from both the recorded and reconstructed images^66,67,106^. The PCI principle is also widely used for quantitative phase imaging by DH^107,108,111^. In particular, Popescu et al. demonstrated white-light diffraction tomography^111^ for imaging microscopic transparent objects, such as live cells. This approach extends diffraction tomography to white-light illumination and imaging rather than scattering plane measurements and was applied to reconstruct the 3D structures of live, unlabeled red blood cells and achieved comparable results to confocal and scanning electron microscopic images.

Concerning the engineering of a light source to reduce the speckle noise in DH, a very interesting work was published in 2011 by Cao et al. in ref.^112^, in which the authors demonstrated how random lasers can be adopted to provide low spatial coherence. By exploiting the low spatial coherence of specifically designed random lasers, speckle-free full-field imaging in the setting of intense optical scattering can be achieved^51^. Choi and co-workers, who developed an off-axis quantitative phase microscopic system that relies on a light source with extremely short spatial coherence length, achieved impressive results as well. The system was able to reduce the diffraction noise while enhancing the spatial resolution^113^.

**Fig. 3:**
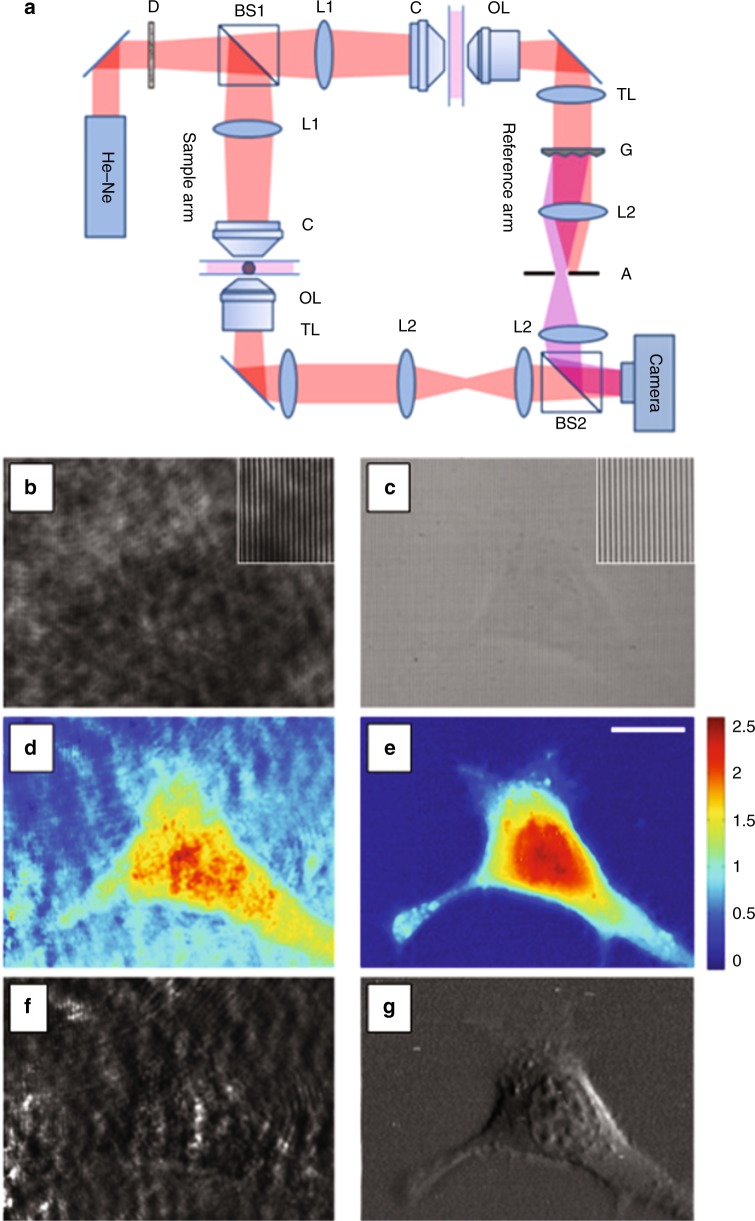
Classical off-axis DH configuration with coherent illumination versus dynamic speckle illumination. **a** Off-axis Mach–Zehnder interferometer. G is the diffraction grating. **b**–**g** Quantitative phase imaging of a live cell using the method that was proposed in ref.^102^: **b** raw interference image, **d** processed quantitative phase image, and **f** numerically simulated DIC image for coherent illumination. **c**, **e**, **g** are the same as **b**, **d**, **f** but with dynamic speckle illumination. The insets in **b**, **c** are magnified images of the background by threefold. Scale bar, 10 μm. Color bar, phase in radians. Reprinted with permission from ref.^113^ [OSA The Optical Society]

Their strategy was to employ a diffraction grating in the reference beam path that generates fine interference fringes with high contrast across the entire field of view using a light source of very low spatial coherence. A striking comparison between holographic reconstructions obtained by using the classical off-axis DH configuration with coherent illumination and with dynamic speckle illumination is reported in Fig. 3.

The last category of optical methods for noise reduction is named multi-look DH (MLDH) and refers to all the DH imaging methods that exploit multiple recordings of the object (i.e., the *looks*) that are captured under various realizations of the noise process^32,33,35,43,52–58,69,73^. In particular, the recording set-up is adjusted to provide noise diversity while keeping the signal highly correlated. Hence, multiple DH reconstructions can be suitably combined to obtain a significant noise contrast reduction. This can be quantified by means of the previously introduced speckle contrast *C*. This performance metric is expected to reduce as a denoising result, and it is bounded by the ideal curve $1/\sqrt L$, where *L* is the number of looks^35^. In such a case, the ideal trend would be obtained in the case of fully uncorrelated noise among the realizations of each pixel, which is not feasible in practice. All the methods that have been proposed so far approximate the performance of the ideal curve and reach saturation values that depend on the extent of the noise decorrelation among multiple DH recordings. In all these cases, any pixel is combined with the homologous pixels in the acquisition stack, so that resolution is preserved at the cost of set-up complexity and dilated recording times. Noise diversity can be obtained by exploiting the decorrelation that exists among channels or introducing additional optical elements into the set-up to achieve time variability of the speckle spot intensities and positions. Using a moving diffuser is perhaps the most intuitive way to introduce time variability into the path that is experienced by the object or the reference beam. Creating variable phase delays changes the resultant of the coherent sum at each sensor element. In turn, multiple realizations of the same pixel can be achieved and the MLDH algorithm can benefit from the introduced redundancy.

Rotating or shifting rough diffusers has been widely adopted within this scope^35,43,69^. Similarly, noise diversity can be provided by slightly changing the illumination angle (gain in *C* of up to 41.7%)^55,70^ or shifting the recording device (gain in *C* of up to 76.9%)^57^. Alternative strategies include employing piezoelectric actuators or spatial light modulators to introduce time-variable phase delays into one of the two interfering wavefronts (gain in *C* of up to 81.1%)^71^. An efficient way to provide speckle diversity is to tune the illumination source to exploit the diversity among multiple channels. Multiple wavelengths were exploited in ref.^42^. An effective multi-channel method for providing noise diversity in the optical set-up is to record holograms by changing the linear polarization of the object beam while using a circularly polarized reference wavefront^72,73^. Another approach consists of moving apertures with areas that are characterized by different transmission factors (in amplitude and/or phase) that can be employed to obtain noise diversity during the exposure time of the recording device^74,75^. However, constraints that are related to the moving aperture implementation limit the number and variety of transmission masks and, in turn, the maximum achievable gain.

As mentioned above, MLDH techniques also require numerical processing to combine the multiple reconstructions to achieve noise reduction. Typically, this is performed by a straightforward average sum (AS):

4 $${\mathbf{A}} = 1/L\mathop {\sum}\nolimits_i {\bf{A}} _i$$

where **A**_*i*_, for *i* = 1,…,*L*, are the amplitude reconstructions of the *L* recorded holograms of the same object. Therefore, MLDH allows a denoised visualization only in numerical reconstruction (either of the amplitude or phase), but it does not give the opportunity to directly synthesize a new denoised digital hologram. In ref.^35^, Memmolo et al. demonstrated a novel encoding method for directly combining multiple digital holograms. The aim was to achieve image improvement in numerical as well as optical reconstruction. The proposed encoding formula was related to the geometric sum of amplitude reconstructions, as reported below.

5 $${\mathbf{A}} \approx \frac{1}{L}\left( {L!\mathop {\prod}\limits_{i = 1}^L {{\mathbf{A}}_i} } \right)^{\frac{1}{L}}$$

**Fig. 4:**
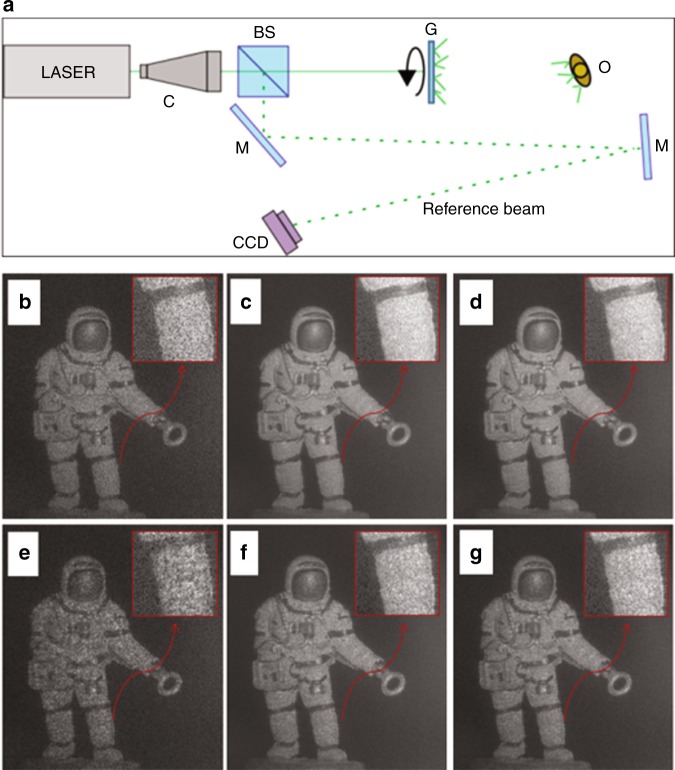
Encoding holograms for speckle-noise reduction. **a** Set-up for hologram recording; G is the rotating ground glass that is used for multiple recordings with noise diversity. **b**–**g** Amplitude reconstructions in the cases of thin (**b**) and large (**e**) speckle grains; **c**, **f** are the results of the AS method, while **d**, **g** are the results that are obtained by using the encoding method. Adapted with permission from ref. ^35^ [OSA The Optical Society]

By the factorization in Eq. (5), it becomes possible to apply the convolution property of the Fourier transform, so that the AS of the reconstructions in the propagation plane reduces to a convolution between holograms in the acquisition plane^35^. The equivalence in Eq. (5) represents a comparison of two encoding approaches. In other words, it is possible to compare them by evaluating the difference in visual quality improvement that is obtainable using the left-hand and right-hand sides of Eq. (5). The analysis that is carried out in ref.^35^ indicates an agreement between the two encoding formulas of >98%. In Fig. 4, we report the comparison between the two encoding formulas and single reconstructions of an astronaut puppet in the cases of two experimental noise levels, which are referred to as “thin” (Fig. 4b) and “large” (Fig. 4e) speckle grains, respectively. In particular, Fig. 4c, f report the result that is achievable by using the AS, while the synthesis that is performed using Eq. (5) is shown in Fig. 4d, g. It is apparent that both methods achieve an ML gain with respect to the SL reconstructions. Moreover, no significant differences are found between the AS and the encoding that is described by Eq. (5), with the latter having the above-mentioned advantage.

## Numerical approaches to noise reduction

Spatial filtering is the most commonly applied numerical method in digital holographic image processing^102,103^. Among the proposed techniques, two main categories can be identified: Bayesian and non-Bayesian strategies. Statistical approaches try to improve the quality of holograms by exploiting knowledge about the noise statistics. For Gaussian and Poisson random processes, if the statistics are at least of the second order, the denoising approaches can be considered Bayesian. This knowledge can be assumed or obtained from data. Existing studies try to model the noise in DH as having Gaussian^64^ or independent Poisson^65^ distributions and use other enforcing priors (e.g., sparsity constraints). However, these often lead to inaccurate estimates, thereby resulting in the creation of severe artifacts or removal of image fine structures. Optimization of acquisition set-up in the sense of imaging resolution can partially overcome these drawbacks, thereby yielding approximations that are closer to the actual conditions^66,67^.

**Fig. 5:**
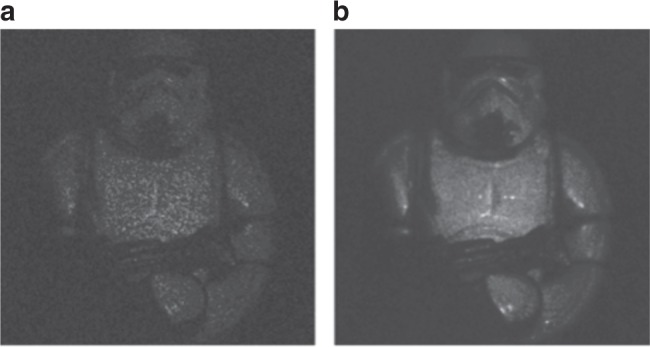
Discrete Fourier filtering. **a** Original noisy reconstruction. **b** Denoised reconstruction after applying the discrete Fourier filtering technique. Reprinted with permission from ref.^41^ [OSA The Optical Society]

By considering approaches that infer the noise statistics from data, a dichotomy can arise. Formally, how the statistics are inferred should not be a discriminating factor. However, in practice, only the approaches that infer statistics from multi-images are referred to as Bayesian, whereas those that use a single shot are not. Although this common taxonomy could be questioned, it is worthwhile to use it to be consistent with the prominent literature of the field. Chen and Li reported in ref.^68^ an interesting example of a Bayesian approach. A digital image denoising technique is applied to multiframe superposed images in terahertz DH. The noise-suppression problem is treated as a Bayesian least-squares estimation and it is solved using Markov chain Monte Carlo sampling. In this algorithm, a weighted mean filter with a Gaussian kernel is first applied to the noisy image. Then the contrast of the image is restored to the former level by nonlinear contrast transform. A more general method is proposed by Leo et al. in ref.^37^ that takes into account not only spatial information but also temporal statistics that are associated with the pixels. A mixture of Gaussian distribution models is used to characterize the observed noise in each pixel. When no complete second-order characterization is available, non-Bayesian strategies must be adopted. Among them are some of the MLDH approaches that are described above in which multiple looks are not optically recorded but hybrid techniques are used. These are used to generate multiple reconstructions with almost-uncorrelated noise, starting from a single-shot DH recording^32,39^. To achieve this important goal, the numerical reconstruction step must be properly modified or additional filters must be introduced that obtain multiple realizations of the noisy reconstruction by tuning their inner parameters. This paradigm has been successfully exploited, thereby showing that digital filters can simulate the action of moving diffusers. Recently, quasi-static colloidal solutions^114^, flowing biological samples^115,116^, and even live microorganisms^117^ have been demonstrated to provide remarkable noise decorrelation. This has been exploited either to improve the performance of optical systems^116,118^ or to allow one to see through turbidity in DH transmission microscopic configurations^114–117^. Milk colloids that experience Brownian motion, flowing red blood cells, and self-propelling bacteria are good examples in this sense and pave the way for the exploitation of biological material as a useful optical element^119^. Moving-aperture-based methods, which are described in section “Optical solutions for noise reduction,” can also be numerically simulated with high accuracy and without introducing complexity into the recording scheme. A good example in this sense is the work that is reported in ref.^41^, in which a Fourier filtering mask was applied to the hologram spectrum and the mask shape and size were varied to obtain multiple realizations of a noisy object after propagation. Despite its simplicity, this method was shown to be very effective in denoising Fourier holograms, as demonstrated in Fig. 5. To date, several techniques that are based on spatial filters and inherited from the research field of image processing have been successfully employed for the denoising of holographic images. In Table 1, we list the most frequently used ones. Their mathematical descriptions are omitted for brevity; readers can refer to Supplementary Information file for a more detailed description, as well as the highlighted original papers in which they were proposed^37,59,120–146^.

**Table 1:** Most frequently used digital filtering methods and the corresponding references

Filters	Refs.
Median	120
Wiener	120
Lee	37,121
Frost (adaptive Gaussian)	59
Wavelet	122–131
Non-local (NL) means	132–135
Block matching 3D (BM3D)	136–138
2D windowed Fourier transform (WFT2D)	139–144
Anisotropic diffusion	145,146

See Supplementary Information file for details about the listed methods

The current general trend is to design novel and efficient spatial filters to be applied on a single recorded hologram. In subsections “Amplitude reconstruction denoising” and “Phase reconstruction denoising,” we evaluate the performance of the methods that are reported in Table 1, when these are applied on amplitude and phase holographic reconstructions, respectively.

### Amplitude reconstruction denoising

**Fig. 6:**
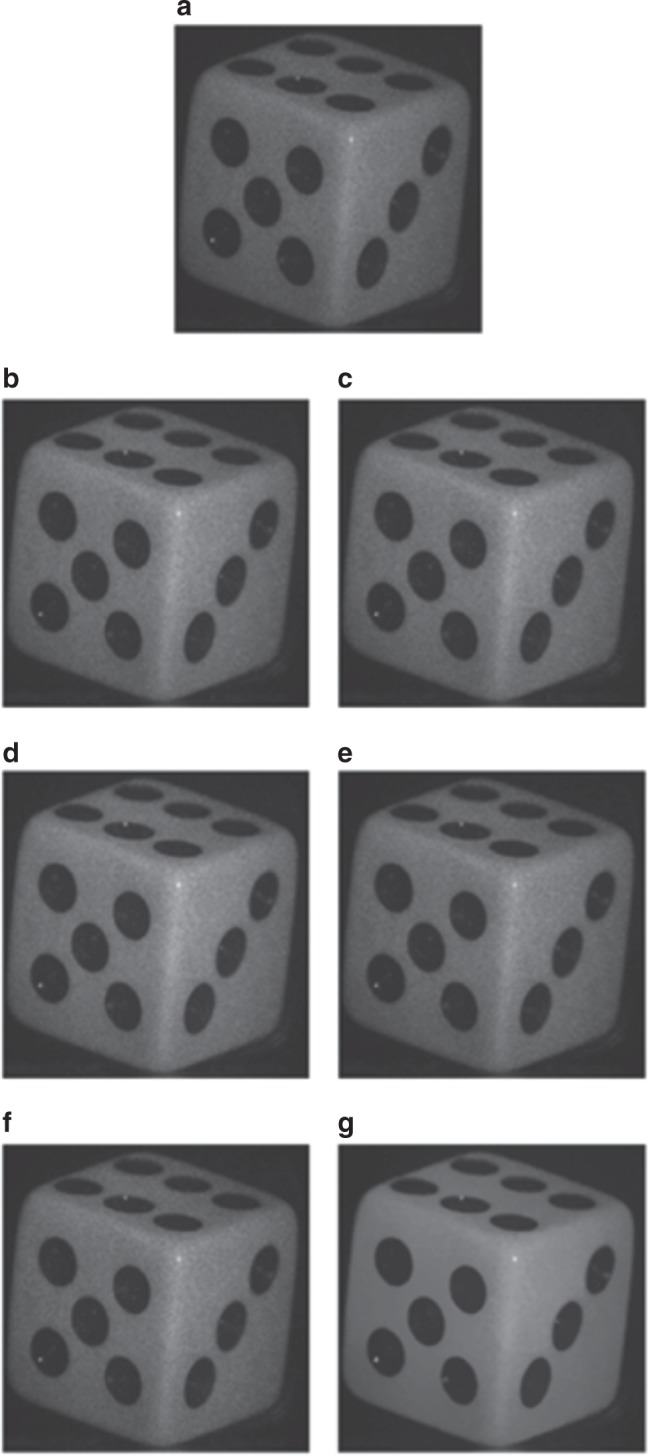
Comparison of speckle reduction filters, which are applied on a dice image that was reconstructed from its digital holographic recording. **a** Back-propagation from the original hologram, **b** denoised with the median filter, **c** denoised with the Frost filter, **d** denoised with the Lee filter, **e** denoised with the bilateral filter, **f** denoised with the wavelet thresholding filter, and **g** denoised with the NL means filter. Reprinted with permission from ref.^60^ [OSA The Optical Society]

The methods that are listed in the previous paragraph were proposed in DH for amplitude reconstruction improvement. Uzan et al. in ref.^60^ reported a very interesting work in which the application of NL means filtering to holographic amplitude reconstructions was proposed and compared with the filters that are commonly used for speckle reduction: the median filter, Lee filter, Frost filter, bilateral filter, and wavelet thresholding. Figure 6 reports this comparison^60^.

Three metrics, namely, Equivalent Number of Looks (ELN), Speckle Suppression Index (SSI), and Speckle Suppression and Mean Preservation Index (SMPI)^147^, are used to evaluate the performance of these methods. The NL means filter performed the best in terms of ELN and SSI, while wavelet thresholding achieved the best performance in terms of SMPI. The key to achieve such remarkable performance is related to the concept of grouping. Grouping means that mutually similar, non-neighboring two-dimensional image blocks are selected and stacked together to form 3D arrays. Then each 3D stack is processed as a whole. The same grouping paradigm is implemented for the BM3D filter, which is recognized as the state of the art in image denoising. BM3D applies shrinkage operators to each stack in a proper 3D transform domain to return filtered versions of the groups^137^.

**Fig. 7:**
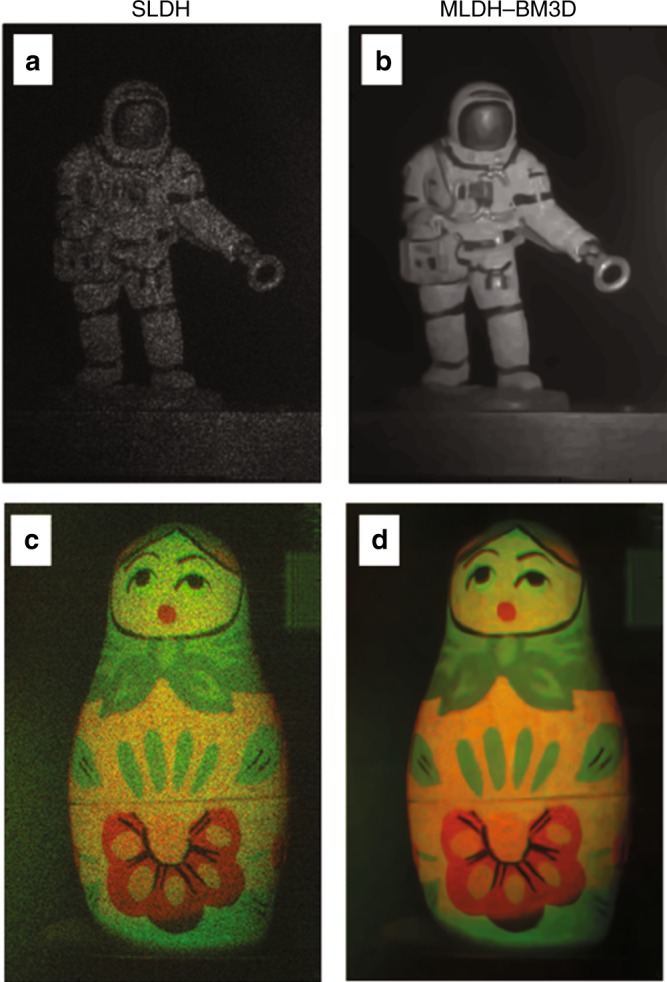
Quasi noise-free DH. Comparison between SLDH reconstructions for single- (**a**) and multi-wavelength (**c**) holograms and the MLDH-BM3D filtered images (**b**, **d**). These images are inspired by the results in ref.^32^

Recently, the relation between the initial signal-to-noise ratio (SNR) of the noisy holographic amplitude reconstruction and the performance of both NL means and BM3D filters have been investigated^32^, revealing that, in the absence of prior information on the noise statistics (non-Bayesian approaches), a low SNR can lead to incorrect grouping and severely affect the reconstruction quality. In such cases, a preliminary filtering would be desirable^137^. This aspect has been investigated by Bianco et al. in ref.^32^, in which it was demonstrated that, by combining the concepts of multi-look, grouping, and collaborative filtering, it is possible to achieve quasi noise-free DH reconstructions. The proposed approach is referred to as MLDH-BM3D and surpasses the limits of both MLDH and BM3D. Indeed, the enhanced grouping algorithm is demonstrated to impose better initial conditions for the subsequent collaborative filtering steps. The BM3D performance is improved as the probability of incorrect grouping is drastically reduced. In addition, MLDH-BM3D is demonstrated to outperform the sole MLDH, with the MLDH theoretical improvement bound^39,57,73^ being overcome for the first time due to the sparsity enhancement filtering. In terms of the taxonomy that is adopted in this review paper, MLDH-BM3D can be classified as a hybrid denoising method because it is a mix of smart optical recording techniques and numerical processing, which are necessary for each other to achieve nearly noise-free DH reconstructions. Experimental validation of MLDH-BM3D was provided in the case of visible wavelength recording^32^ (both single and multiple wavelengths) and infrared DH (IRDH)^33^ in very-low-SNR conditions. In Fig. 7, we summarize the main results that are achieved in ref.^32^ for the cases of a single-wavelength recording of an astronaut puppet that is severely corrupted by speckle noise (Fig. 7a) and dual-wavelength recording (i.e., red and green lasers) of a Matryoshka doll, where MLDH-BM3D is applied on both color components (Fig. 7c). Experimental validation of MLDH-BM3D was provided in the case of visible wavelength recording^32^ (both single and multiple wavelengths), and the reconstructions of a single recorded hologram have been labeled as single-look DH images (SLDH), in contrast to the multiple recording that is used for the MLDH step. By evaluating the performance of MLDH-BM3D filtering that is reported in Fig. 7b, d in terms of the percentage of noise suppression^32^, an improvement of up to 98% is achieved, i.e., the quality of the DH reconstructions was comparable to those of low-coherence techniques for the first time.

**Fig. 8:**
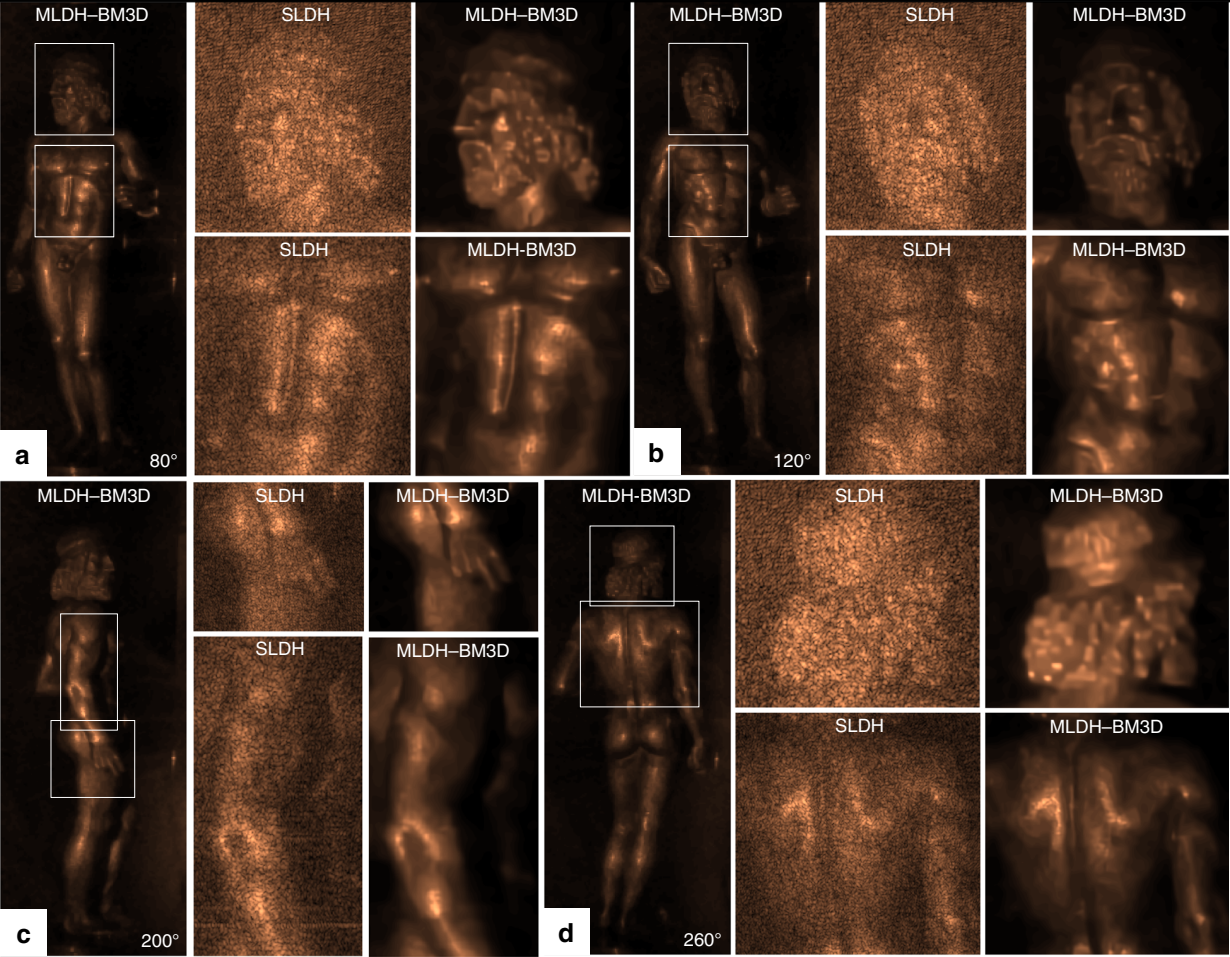
MLDH-BM3D in the case of IRDH images. IR reconstructions of the “Bronzo di Riace” without and with MLDH-BM3D processing with the aim of comparing the noisy and denoised images at multiple vewing angles: **a** 80°, **b** 120°, **c** 200°, and **d** 260°. Reprinted with permission from ref.^33^ [OSA The Optical Society]

The case of IRDH is very challenging in the framework of noise reduction because the coherent noise level in a long-IR hologram is far larger than that of a visible-wavelength recording, thereby resulting in very poor quality of both numerical and optical reconstructions^33^. Bianco and co-workers demonstrated that MLDH-BM3D is still the best-performing denoising method and achieves noteworthy results. Figure 8 reports the comparison between the SLDH and MLDH-BM3D reconstructions of a famous work of art, namely, the “Bronzo di Riace.” Remarkably, a percentage of total noise suppression of 80% was obtained^33^.

### Phase reconstruction denoising

All the methods that are described above have been recently evaluated in the context of digital holographic interferometry on a benchmark^47,148^. The database for the benchmark consists of simulated fringe images with controlled fringe pattern type and noise level. Five fringe patterns were simulated to introduce a “fringe diversity” and calculate statistics on the obtained results. With the same motivation, five values for the SNR were introduced. Thus 25 noisy phase maps with cosine SNR that varied from approximately 3 to 12 dB were obtained, which well-matched real experimental conditions. The relevant metric for this evaluation is the phase error, which represents the standard deviation of the wrapped phase difference between the denoised and noisy phase maps.

**Fig. 9:**
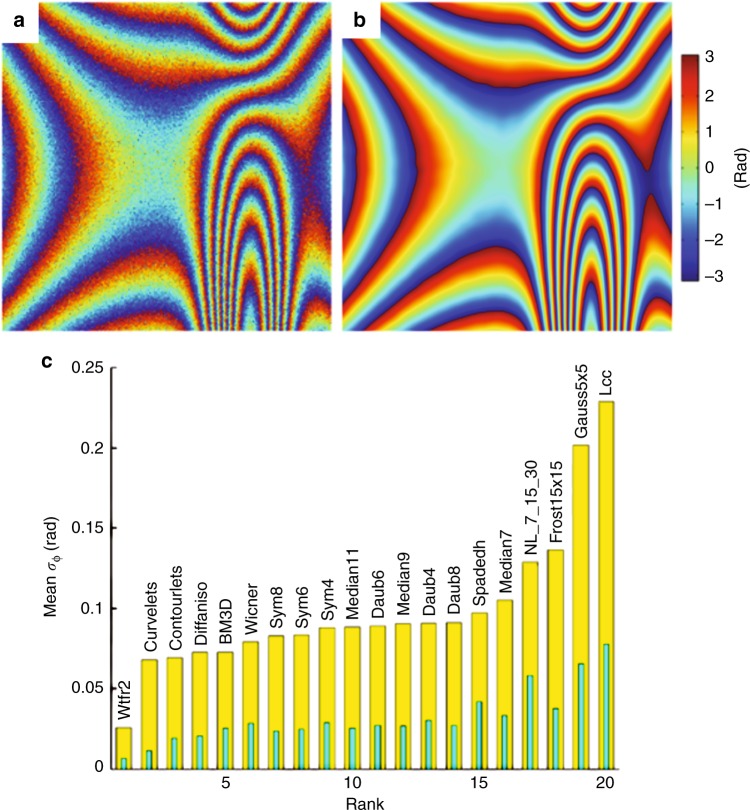
Performance of filtering algorithms for phase denoising. **a** Noisy phase map of a simulated fringe pattern with realistic speckle decorrelation noise, with an input cosine SNR of 3.59 dB, **b** denoised phase map with the windowed Fourier transform method, with the output cosine SNR estimated at 30.11 dB, and **c** ranking of 20 selected denoising algorithms in terms of mean standard deviation of phase error; with an error of approximately 0.03 rad, the windowed Fourier transform method (Wtfr2 in the graph) has the best rank. Adapted with permission from ref.^47^ [OSA The Optical Society]

Figure 9 summarizes the results that were obtained for the phase error metric. The height of each yellow color bar in Fig. 9c represents the average of values over the entire database. We also represent, within each index bar, a second bar of a different color that corresponds to the standard deviation that is associated with the values that are displayed for the entire database. Since the first published ranking^47^, parameters of selected methods have been optimized to increase the filtering performance. This is the case for WFT2F, stationary discrete wavelets (Sym8, Sym6, Sym4, Daub8, Daub6, Daub4), Contourlets, and the Wiener filter. The WFT2F method, which is denoted as Wtfr2 in Fig. 9c, performs the best, with an average value of 0.025 rad, followed by curvelets with 0.07 rad. The BM3D method (known as the state of the art for amplitude imaging) yields equivalent results to curvelets but with a larger variance. Moreover, the ranking indicates the good performance of median filters 9 × 9 and 11 × 11 at 0.09 rad; between 0.08 and 0.09 rad, all stationary wavelet methods exhibit similar variance values. Finally, many performance evaluation metrics can be implemented to compare denoising algorithms^47,60^. They are omitted for brevity, but readers can refer to Supplementary Information file for a description of the most commonly used metrics in DH.

## Conclusions and future key challenges

In this review, we have discussed and classified strategies for speckle noise reduction in DH. Two main classes have been considered: optical and numerical approaches. It is inferred that the main trend within this scope involves the reduction of the source coherence, although the use of multiple recordings of the same target while varying the noise realizations has been widely investigated in the past decade. In addition, several research groups proposed novel numerical approaches that are based on increasingly sophisticated spatial filters. At the end of our tour of the best-performing denoising methods, we tried to identify the gold standard in this deeply investigated research field.

The best optical solution for noise reduction in DH is white-light recording, where holograms and the corresponding reconstructions are intrinsically free from speckle. Hence, this strategy can be considered an a priori solution of the speckle noise problem. Poon et al.^104,105^ and Kim^106^ demonstrated the feasibility of this approach and achieved impressive results in terms of SNR. However, two main drawbacks can be identified: (i) optical arrangements need to be much more robust than the classical coherent holographic imaging systems and (ii) when the SNR in the object reconstruction was compared to theoretical predictions, a decrease in the SNR as the inverse of the number of resolved object pixels was observed, and some discrepancies can be revealed and explained by imperfections in the optics. These two issues were investigated 30 years ago by Ribak et al.^149^ and are still relevant today. The same limitations can be recognized for MLDH methods, where the theoretical bound on noise decorrelation between the multiple looks inherently restricts the achievable improvement in terms of SNR^39^.

In contrast, novel or modified versions of existing numerical approaches for noise reduction in DH are continuously published, thereby demonstrating an ever-growing interest in this research field worldwide. Our review has tried to consider almost all the main contributions of the past decades by focusing on critical papers that also provide comparisons between the existing and high-performing denoising strategies. We recognized that BM3D, which was proposed by Dabov et al.^136,137^, is the best spatial filter for holographic amplitude reconstructions with suitable starting SNR and additive Gaussian noise. However, for a strong initial noise level and multiplicative noise, Bianco et al. demonstrated that the hybrid method that is obtained by the joint action of MLDH and BM3D^32,33^ can reach up to 98% noise suppression. Strategies for phase reconstruction denoising have been evaluated in the context of digital holographic interferometry on a benchmark by Montresor and co-workers^47,148^. Among the 20 selected denoising algorithms that were compared in terms of mean standard deviation of the phase error, the best was the WFT2F method, which was proposed by Kemao^139^, for the case of simulated fringe patterns. In the case of more-structured objects (i.e., with more complex features to be preserved) or larger image size, the power of learning/stacking methods (e.g., BM3D) is expressed in full. Hence, these are expected to perform slightly better. Moreover, the relative ranking among the various wavelet bases is expected to change depending on the content of the object frequencies. Although this research field may seem to be saturated, a unified framework for noise suppression in DH that is capable of working efficiently for both amplitude and phase reconstructions has not been deeply investigated. Very few denoising strategies have been proposed that are specifically suited to denoise reconstructed images from digital holograms. Among them, the sparsity denoising of digital holograms method is designed to reduce the noise in DH operating on the reconstructed complex field. Thus it works on both amplitude^38^ and phase^40^ reconstructions. Very recently, an extension of the BM3D framework to complex observables was introduced^150,151^, and it is shown that complex domain BM3D outperforms the separate denoising of amplitude and phase by means of a BM3D algorithm, as well as the separate denoising of the real and imaginary parts of complex-valued data.

## Electronic supplementary material

Supplementary information
