# Supplementary material for: Strategies for reducing speckle noise in digital holography
Source: Light Sci Appl. 2018 Aug 1;7:48. doi: 10.1038/s41377-018-0050-9 (PMC6106996; doi:10.1038/s41377-018-0050-9)
Supplement: Supplementary file 1 — Supplementary information [file 41377_2018_50_MOESM1_ESM.docx]

**Supplementary Information**

**Strategies for reducing speckle noise in digital holography**

Vittorio Bianco,^1^ Pasquale Memmolo,^1,*^ Marco Leo,^1^ Silvio Montresor,^2^ Cosimo Distante,^1^ Melania Paturzo,^1^ Pascal Picart,^2^ Bahram Javidi,^3^ and Pietro Ferraro.^1^

^1^CNR-ISASI Istituto di Scienze Applicate e Sistemi Intelligenti “E. Caianiello”, via Campi Flegrei 34, 80078 Pozzuoli (NA), Italy.

^2^Université du Maine, CNRS UMR 6613, LAUM, Avenue Olivier Messiaen, 72085 Le Mans Cedex 9, France.

^3^Electrical and Computer Engineering Department, University of Connecticut, U-4157, Storrs, Connecticut 06269, U.S.A.

*Corresponding author: [p.memmolo@isasi.cnr.it](mailto:p.memmolo@isasi.cnr.it)

**A: Working principle of the digital filter methods listed in Table 1**

The most pursued goal in the field of denoising DHs is to find novel and efficient spatial filters to be applied on one single recorded hologram. The most used ones, reported in Table 1 of the main text, are summarized here. Several types of kernels can be considered: the moving average filter, the median filter which is efficient to remove impulsive noise and the Gaussian filter. These filters are generally very easy to be implemented and they constitute reference methods to evaluate other more complex algorithms.

In image processing, the Wiener method is a filtering operation which aims at producing an estimate of a noise free target image by using a spatially invariant linear filter.^1^ From the mathematical point of view, the **Wiener filter** is the solution of the least square minimization of the difference between the original noise free target image and the filtered image in case of a linear spreading model with additive noise. As the goal is only to reduce the impact of the noise, the spreading function can be chosen as unitary, so in this case, the transfer function of the Wiener filter^1^, *W*(*u*,*v*), is given by Eq.(1):

$W\left(u,v\right)=\frac{\Phi_{s}\left(u,v\right)}{\Phi_{s}\left(u,v\right)+\Phi_{b}\left(u,v\right)},$ (1)

where Φ*_s_* and Φ*_b_* are respectively the power spectrum density of the original image and the power spectrum density of noise. These quantities are not known a priori so they have to be estimated.

Synthetic Aperture Radar (SAR) filtering methods can be also applied to DH speckle images. Indeed, DHs and SAR data share similar noise properties, the noise model being considered as multiplicative. A good example is the adaptive **Lee filter**,^2^ in which the parameters are tuned according to the local statistical properties of the image to be processed. The parameters are estimated in a neighborhood of the pixel for which one aims at restoring the correct value. The filtering can be mathematically described by:

$d\left( x,y \right)=\alpha s\left( x,y \right)+\left( 1-\alpha\right)\mu_{s}=\mu_{s}+\alpha[s\left( x,y \right)-\mu_{s}].$ (2)

In Eq.(2), the parameter $\alpha={\sigma_{s}^{2}}/{(\sigma_{s}^{2}+\sigma_{b}^{2})}$ depends on the ratio between the signal variance in the considered local window, $\sigma_{s}^{2},$and the sum between $\sigma_{s}^{2}$ and the speckle variance, $\sigma_{b}^{2}$. In a region characterized by homogeneous values ($\sigma_{s}^{2}\ll\sigma_{b}^{2}$), the local variation coefficient is low (α≈0), and the filter locally smooths the image thus reducing noise. On the opposite, in regions characterized by high local variations like edges and fine details ($\sigma_{s}^{2}\gg\sigma_{b}^{2}$) α approaches unity and the current value of the pixel is considered, thus preserving the resolution.

**Decomposition on wavelet basis** and thresholding was extensively considered for image enhancement.^3,4^ The principle is to use a threshold on the wavelet coefficients computed from the image to be enhanced. When the wavelet coefficient space of the signal is sparse, the highest coefficients concentrate information about the image. It follows that applying a threshold eliminates the least significant coefficients. These coefficients are supposed to be related to noise. As a general rule, the model considers an additive noise. When considering the wavelet transformation, the same process is applied to the coefficients. In the case where the noise is multiplicative, computing the logarithm of the signal permits to deal with additive signal and noise. For example, such an approach was proposed for noisy SAR images.^5^ Several parameters can be adjusted to optimize wavelet-based denoising: wavelet basis, number of decomposition levels, profile of the thresholding operator, threshold values and the way the threshold is estimated. Various wavelet bases have been proposed, like *Daubechies* wavelets and *symlets* built from the tensor product of their one dimensional release, and other *curvelets* and *contourlets*,^6,7^ built in a circular paving plane. Such bases offer a wide diversity of waveforms. In the past, noisy speckle interferograms were successfully processed using such approaches.^8-12^

Alternatives for enhancement of images corrupted by additive noise were proposed exploiting the **Non Local Means** (NLM) method.^13-16^ The idea is to replace the pixel value by a weighted sum of values included in patches. The patches are chosen in the neighborhood of the processed pixel. The correlations between the local patch of the pixel to be processed and the neighborhood patches that are taken into account provide the weighting. Thus, when a patch is highly correlated with the local patch of the pixel to be processed, the pixel is taken into account in the weighting with a coefficient evaluated from a Gaussian kernel. The kernel has for argument the Euclidean distance between the two patches.

Based on a similar idea, the transform-based **Block-Matching 3-D (BM3D)** filter proposed by K. Dabov was established as the state-of-the art in image de-noising.^17-19^ The BM3D filter efficiently combines different approaches and synthesizes major advances of the recent years.^18^ Among them, image decomposition in patches (similarly to the NL-means) and use of shrinkage operators applied on image transform domain as wavelet representations are worth to be mentioned. The basic principle of the BM3D filter is to consider nonlocal image modelling by grouping and collaborative filtering. Grouping means that mutually similar 2-D image patches are detected and stacked together in 3-D. By definition, the pixels belonging to a group are highly correlated (both inter-segment and intra patch correlation of the pixels occur inside each group). Thus, a very sparse representation of the group is allowed in suitable 3D transform domains, which can be exploited to discriminate between signal and noise components.^17-19^ In other words, the group is 3D transformed, then coefficient thresholding is applied to retain the sole signal components. Finally, inverse 3D transform of the group is taken. Since an image segment is allowed to belong to multiple groups, multiple estimates of each segment are obtained, which improve the estimate (aggregation procedure^17-19^).

In order to take into account the non-stationary property of the speckle noise, the **2-D windowed Fourier transform filter** **(WFT2F)** method was proposed by Q. Kemao.^20-25^ It is based on a local Fourier transform (FT) which permits to extract useful frequency components from noise with more localization accuracy than with a FT computed overall. As like wavelet transforms, the filtering process uses a threshold for the modulus of the WFT2F coefficients and the phase is unchanged. The WFT2F can be interpreted as a projection of the input image onto 2-D oscillating functions which are localized both in the spatial and the frequency domain by means of a modulated Gaussian kernel. A hard thresholding applied to coefficients provides the filtering. Finally an IWFT2F is applied on the filtered coefficients in order to get the de-noised image. The phase can be extracted from the output complex image with the help of an arctangent operator to get the modulo 2π phase of the fringe pattern.

**Anisotropic diffusion** is as an adaptive technique which provides a low-pass filtering while preserving edges that are included inside the image to be processed.^26,27^ It is based on scale space theory. Each iteration is equivalent to make a step forward in the scale space. At every step, the resulting image loses resolution, as high spatial frequencies which are characteristic of the speckle noise that one wants to filter out are gradually eliminated. On the opposite to a basic filter, the main idea that lies within anisotropic diffusion is to estimate the edges on the image. The filtering occurs along the edges and not across them. So estimating them is crucial for this algorithm. A good estimator would theoretically be equal to zero within a homogeneous area and proportional to the local contrast beside an edge. In practice, the gradient operator is well known to provide a very good estimation of the edges. Taking into account this estimator and going one step forwards in the scale space leads to the use of the equation of anisotropic diffusion of heat. For numerical calculation, Perona et al. suggested two models of function that permit the calculation of the conduction coefficient that gives a very good estimation of the speckle noise as it favors high contrast edges over low contrast ones.^26^

**B: Performance evaluation metrics**

As a general rule, and whatever the targeted application, metrics are the main part of any quantitative evaluation of algorithms designed to reduce noise. In a recent paper^28^, a review of the metrics that can be used in the domain of digital holography was provided. We invite the reader to have a look at the paper and to the detailed explanations about metrics. In this section, we remind the most significant results about metrics.

A large variety of metrics is based on the concept of signal-to-noise ratio (SNR). For example, the measurement of the evolution of this ratio before and after processing yields the metric “gain of SNR”, defined by *G_SNR_*^29^:

$G_{SNR}=O_{SNR}-I_{SNR},$ (3)

with

$O_{SNR}=10\log10\left[\frac{\sum_{i,j} s\left(i,j\right)^{2}}{\sum_{i,j} \left(s\left(i,j\right)-d\left(i,j\right)\right)^{2}}\right],$ (4)

$I_{SNR}=10\log10\left[\frac{\sum_{i,j} s\left(i,j\right)^{2}}{\sum_{i,j} \left(s_{n}\left(i,j\right)-s\left(i,j\right)\right)^{2}}\right].$ (5)

In these equations, *s*(*i*,*j*), *s_n_*(*i*,*j*) and *d*(*i*,*j*) represent respectively the noise free reference image, the noisy image, and the restored image. A strictly equivalent approach named “improved SNR” is proposed in Refs. [30,31]. The most used metric in the image processing community is, without any contest, the maximum SNR (“peak SNR”), *P_SNR_ ,* defined for images digitized with 8 bits by:

$P_{SNR}=10\log10\left[\frac{255^{2}}{\frac{1}{MN}\sum_{i,j} \left(s\left(i,j\right)-d\left(i,j\right)\right)^{2}}\right],$ (6)

where MxN is the total number of pixels. For potential applications in the field of contact-less metrology dealing with phase measurements, the metric named “phase error”^29-38^ is more suited. Its main advantage is that it provides a quantitative output to evaluate the error on the measured data. The phase error is defined by:

$TPE=\sqrt{\frac{1}{MN}\sum_{i,j} (s(i,j)-d(i,j))^{2}-\left(\frac{1}{MN}\sum_{i,j} (s\left(i,j\right)-d(i,j)\right)^{2}},$ (7)

TPE stands for “true phase error”. In Ref. [28] is established a close link between the *P_SNR_* and the TPE. The relation between these two metrics is given by:

$TPE\cong\pi e^{\left(-\log\left(10\right)\times P_{SNR}/20\right)}.$ (8)

In this equation, *P_SNR_* has to be evaluated on the cosine of the wrapped phase, and not on the raw phase. The illustration is shown in Supplementary Figure S1. The red curve corresponds to the equation and the blue curve to the result of a benchmark with 37 algorithms and 25 phase fringe patterns^28,29^.


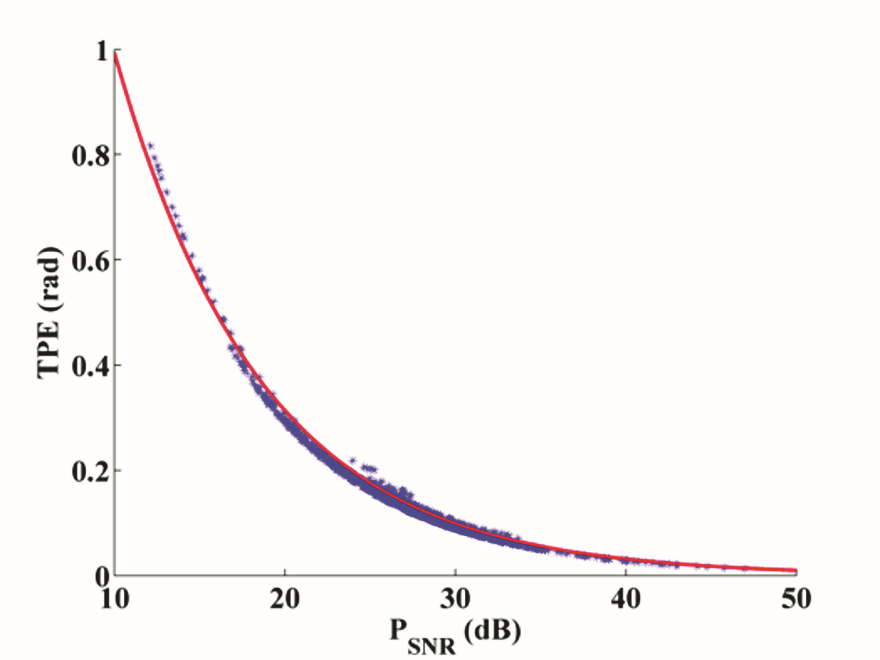


Fig. S1. Relation between *P_SNR_* and *TPE*. Red curve: the algebraic relation. Blue curve: simulation results for 37 algorithms and 25 phase fringe patterns^28,29^. Reprinted with permission from ref. [28], [OSA – The Optical Society].

In the case of data from experiments, it is quite seldom to obtain a noise free reference image. Thus, it is of interest to consider metrics that can be calculated by considering the noisy and de-noised images. This is the case, for example, for the metrics proposed in Refs. [29,32] and named the “signal-to-distortion-ratio” (SDR). Unfortunately, this metric has a weak correlation with metrics using a noise free reference image. It follows that the results of rankings obtained with the SDR are quite different than those obtained with metrics with reference images, as was demonstrated for a database constituted with realistic simulations of noisy phase fringe patterns^29^. To overcome such limitation, a novel metric was proposed in Ref. [28], and is called EPE, as “estimated phase error”. The principle is that the estimation of the most probable noise free reference image is calculated using a reference algorithm. The proposal is to use the 2-D dimensional windowed Fourier transform as the reference algorithm which constitutes the state of the art for de-noising phase images^29^.

As a concluding remark of this section, the metrics calculated on quadratic errors, as presented, do not take into account of distortions of the structures in the phase fringe patterns. Then, a lot of metrics related to perception were proposed in the past^28^. The most used perception metric is the quality index^39^:

$Q_{index}=\frac{\sigma_{sd}}{\sigma_{s}\sigma_{d}}\frac{2\mu_{s}\mu_{d}}{\mu_{s}^{2}+\mu_{d}^{2}}\frac{2\sigma_{s}\sigma_{d}}{\sigma_{s}^{2}+\sigma_{d}^{2}}.$ (9)

In this equation, *µ_s_* and *µ_d_* are the mean values of images *s*(*i*,*j*) and *d*(*i*,*j*), *σ_s_* and *σ_d_* are their variances and *σ_sd_* their covariance. This metric indicates the quality of a perceived image for any observer. This metric exhibits strong correlations with TPE and P_SNR_ ^28,29,39^. It follows that rankings are similar.

References

1. Gonzales R C, Woods R E. *Digital image processing*. (3rd Ed.). Upper Saddle River: Prentice Hall; 2008.
2. Lee J S. Digital image enhancement and noise filtering by using local statistics. *IEEE Trans. on Patt. Anal. And Mach. Intell.* 1980; **2**: 165-168.
3. Mallat S. *A wavelet tour of signal processing*. New York: Academic Press; 1999.
4. Donoho D L. De-noising by soft-thresholding. *IEEE Trans. on Inf. Theory* 1995; **41**: 613-627.
5. Xie H, Pierce L E, Ulaby F T. Sar speckle reduction using wavelet denoising and markov random field modelling. *IEEE Trans. Geosci. Remote Sens.* 2002; **40**(10): 2196-2212.
6. Starck J L, Candès E J, Donoho D L. The curvelet transform for image denoising. *IEEE Trans. Image Proc.* 2002; **11**(6): 670-684.
7. Do MN, Vetterli M. The contourlet transform: an efficient directional multiresolution image representation. *IEEE Trans. Image Proc.* 2005; **14**(12): 2091-2106.
8. Frederico A, Kaufmann G H. Denoising in digital speckle pattern interferometry using wave atoms. *Opt. Lett.* 2007; **32**: 1232-1234.
9. Kaufmann G H, Galizzi G E. Speckle noise reduction in television holography fringes using wavelet thresholding. *Opt. Eng.* 1996; **35**(1): 9-14.
10. Shulev A A, Gotchev A, Foi A, Roussev I R. Threshold selection in transform-domain denoising of speckle pattern fringes. *Proc. SPIE.* 2006; **6252**: 625220.
11. Barj E M, Afifi M, Idrissi A A, Nassim K, Rachafi S. Speckle correlation fringes denoising using stationary wavelet transform. Application in the wavelet phase evaluation technique. *Opt. Laser Technol.* 2006; **38**: 506-511.
12. Bang L T, Li W, Piao M, Alam M A, Kim N. Noise reduction in digital hologram using wavelet transforms and smooth filter for three-dimensional display. *IEEE Photon. J.* 2013; **5**(3): 6800414.
13. Buades A, Coll B, Morel J M. A review of image denoising algorithms, with a new one. *Multiscale Model. Simul.* 2005; **4**(5): 490-530.
14. Buades A, Coll B, Morel J. A non-local algorithm for image denoising. *Proc. IEEE Computer Society Conference on Computer Vision and Pattern Recognition* 2005; **2**(2): 60-65.
15. Deledalle C, Denis L, Tupin F. NL-InSAR: Non-Local Interferogram Estimation. *IEEE Trans. on Geoscience and Remote Sensing* 2011; **49**(4): 1441-1452.
16. Coupé P, Hellier P, Kervrann P, Barillot C. Nonlocal means-based speckle filtering for ultrasound images. *IEEE Trans. Image Proc.* 2009; **18**(10): 2221-2229.
17. Dabov K, Foi A, Katkovnik V, Egiazarian K. Image denoising with block-matching and 3D filtering. *Proc. SPIE* 2006; 6064A-30.
18. Dabov K, Foi A, Katkovnik V, Egiazarian K. Image denoising by sparse 3D transform-domain collaborative filtering. *IEEE Trans. Image Proc.* 2007; **16**(8): 2080-2095.
19. Katkovnik V, Foi A, Egiazarian K, Astola J. From local kernel to nonlocal multiple-model image denoising. *Int. J. Computer Vision* 2010; **86**(1): 1-32.
20. Kemao Q. Windowed Fourier transform for fringe pattern analysis. *Appl. Opt.* 2004; **43**: 2695-2702.
21. Huang L, Kemao Q, Pan B, Asundi A. Comparison of Fourier transform, windowed Fourier transform, and wavelet transform methods for phase extraction from a single fringe pattern in fringe projection profilometry. *Opt. & Las. Eng.* 2010; **48**(2): 141-148.
22. Kemao Q, Nam L, Feng L, Soon S. Comparative analysis on some filters for wrapped phase maps. *Appl. Opt.* 2007; **46**: 7412-7418.
23. Kemao Q. On window size selection in the windowed Fourier ridges algorithm. *Opt. & Las. Eng.* 2007; **45**(12): 1186-1192.
24. Kemao Q. Two-dimensional windowed Fourier transform for fringe pattern analysis: Principles, applications and implementations. *Opt. & Las. Eng.* 2007; **45**(2): 304-317.
25. Yatabe K, Oikawa Y. Convex optimization based windowed Fourier filtering with multiple windows for wrapped phase denoising. *Appl. Opt.* 2016; **55**: 4632-4641.
26. Perona P, Malik J. Space scale and edge detection using anisotropic diffusion. *IEEE Trans. on Pat. Anal. Mach. Int.* 1990; **12**(7): 629-639.
27. Gerig G, Kubler O, Kikinis R, Jolesz F A. Nonlinear anisotropic filtering of MRI data. *IEEE Transactions on Medical Imaging* 1992; **11**(2): 221-232.
28. Montresor S, Picart P, Karray M. Reference-free metric for quantitative noise appraisal in holographic phase measurements. *J. Opt. Soc. Am. A* 2018; **35**, A53-A60.
29. Montresor S, Picart P. Quantitative appraisal for noise reduction in digital holographic phase imaging. *Opt. Express* 2016; **24**(13), 14322-14343.
30. Boucas-Dias J, Katkovnik V, Astola J, Egiazarian K. Absolute phase estimation: adaptive local denoising and global unwrapping. *Appl. Opt.* 2008; **47**(29), 5358-5369.
31. Yatabe K, Oikawa Y. Convex optimization-based windowed Fourier filtering with multiple windows for wrapped-phase denoising. *Appl. Opt.* 2016; 55(17), 4632-4641.
32. Memmolo P, Iannone M, Ventre M, Netti PA, Finizio A, Paturzo M, Ferraro P. Quantitative phase maps denoising of long holographic sequences by using SPADEDH algorithm. *Appl. Opt.* 2013; **52**: 1453-1460.
33. Bang LT, Ali Z, Quang PD, Park JH, Kim N. Compression of digital hologram for three-dimensional object using Wavelet-Bandelets transform. *Opt. Express* 2011; **19**, 8019-8031.
34. Bang LT, Li W, Piao ML, Alam MA, Kim N. Noise reduction in digital hologram using wavelet transforms and smooth filter for three-dimensional display. *IEEE Photon. J.* 2013; **5**(3), 6800414.
35. Frederico A, Kaufmann GH. Phase retrieval in digital speckle pattern interferometry by use of a smoothed space-frequency distribution. *App. Opt.* 2003; **42**(35), 7066-7071.
36. Shulev AA, Gotchev A, Foi A, Roussev IR. Threshold selection in transform-domain denoising of speckle pattern fringes. *Proc. SPIE* 2006; **6252**, 625220.
37. Frederico A, Kaufmann GH. Comparative study of wavelet thresholding methods for denoising electronic speckle pattern interferometry fringes. *Opt. Eng.* 2011; **40**(11), 2598-2604.
38. Montresor S, Picart P, Sakharuk O, and Muravsky L. Error analysis for noise reduction in 3D deformation measurement with digital color holography. *J. Opt. Soc. Am. B* 2017; **34**(5), B9-B15.
39. Wang Z, Bovik AC, Lu L. Why is image quality assessment so difficult?. *Proc. IEEE ICASSP* 2002; **4**, 3313-3316.
